# E-selectin S128R polymorphism and severe coronary artery disease in Arabs

**DOI:** 10.1186/1471-2350-7-52

**Published:** 2006-06-06

**Authors:** Khaled K Abu-Amero, Olayan M Al-Boudari, Gamal H Mohamed, Nduna Dzimiri

**Affiliations:** 1 Genetics Department, King Faisal Specialist Hospital and Research Centre (MBC – 03), P. O. Box 3354, Riyadh 11211, Saudi Arabia; 2 Department of Biostatics, Epidemiology and Scientific Computing, King Faisal Specialist Hospital and Research Centre (MBC – 03), P. O. Box 3354, Riyadh 11211, Saudi Arabia

## Abstract

**Background:**

The E-selectin p. S128R (g. A561C) polymorphism has been associated with the presence of angiographic coronary artery disease (CAD) in some populations, but no data is currently available on its association with CAD in Arabs.

**Methods:**

In the present study, we determined the potential relevance of the E-selectin S128R polymorphism for severe CAD and its associated risk factors among Arabs. We genotyped Saudi Arabs for this polymorphism by PCR, followed by restriction enzyme digestion.

**Results:**

The polymorphism was determined in 556 angiographically confirmed severe CAD patients and 237 control subjects with no CAD as established angiographically (CON). Frequencies of the S/S, S/R and R/R genotypes were found as 81.1%, 16.6% and 2.3% in CAD patients and 87.8%, 11.8%, and 0.4% in CON subjects, respectively. The frequency of the mutant 128R allele was higher among CAD patients compared to CON group (11% vs. 6%; odds ratio = 1.76; 95% CI 1.14 – 2.72; *p *= .007), thus indicating a significant association of the 128R allele with CAD among our population. However, the stepwise logistic regression for the 128R allele and different CAD risk factors showed no significant association.

**Conclusion:**

Among the Saudi population, The E-selectin p. S128R (g. A561C) polymorphism was associated with angiographic CAD in Univariate analysis, but lost its association in multivariate analysis.

## Background

E-selectin (endothelial leukocyte adhesion molecule; ELAM1) is an 11-kD cell surface glycoprotein expressed on endothelial cells after activation by cytokines, and mediates adhesion of circulating monocytes and lymphocytes to endothelial cells. This adherence to activated arterial endothelium is one of the earliest detectable events in the pathogenesis of atherosclerosis [1]. Double-knockout mouse experiments suggested that E-selectin plays an essential role in both early and advanced stages of atherosclerotic lesion development and that mutations in cellular adhesion molecules like E-selectin may act as genetic risk factors for coronary atherosclerosis [2,3]. Additionally, the involvement of E-selectin in cardiovascular diseases is suggested by the fact that it is expressed only in activated endothelial cells. Amino acid change from serine (S) to arginine (R) at codon 128 (S128R), which corresponds to A > C nucleotide change at position 561 (A561C), in the epidermal growth factor-like domain of the E-selectin gene has been implicated in the pathogenesis of CAD in several ethnic groups, including Germans, Japanese, Americans, Chinese and Africans [4-10]. The 128R mutant allele was significantly higher in the CAD patients than in controls (12.6% versus 6.7%, 17.4% versus 7.1%, and 19.5% versus 10.6%) in Japanese, [4] German [11] and white American [6] populations, respectively. However, no previous studies are available on possible association of this polymorphism with CAD among Arabs. Furthermore, apart from a study, which did not find a link between this mutation and CAD in Austrian patients with diabetes mellitus [12], there is hardly any data in the literature pertaining to the possible association of this mutation for the different CAD risk factors. Therefore, the aim of this investigation was to evaluate the potential relevance of E-selectin 128R polymorphism for angiographic CAD and its risk factors in Arabs, using the Saudi population as a study model.

## Methods

### Study population

Two groups of Saudi individuals were recruited for the present study. The patient group comprised 556 candidates (396 males and 160 females; mean age 50 ± 16 yr) of Saudi Arabian descent with angiographically documented severe CAD. The inclusion criterion for CAD was the presence of angiographically determined narrowing of the coronary vessels by at least 70%, which we define as having severe disease. Exclusion criteria for CAD were major cardiac rhythm disturbances, incapacitating or life-threatening illness, major psychiatric illness or substance abuse, history of cerebral vascular disease, neurological disorder, and administration of psychotropic medication. A second group of 237 individuals (105 males and 132 females, mean age 50 ± 17 yr) undergoing surgery for heart valvular diseases and those who reported with chest pain, but were established to have no significant coronary stenosis by angiography, were recruited as angiographed controls (CON). Exclusion criteria for this group included among others diseases such as cancer, autoimmune disease, or any other disorders likely to interact with variables under investigation. This study was performed in accordance with the regulations laid down by the Hospital Ethics Committee and all participants signed an informed consent.

### DNA preparation

Five ml of peripheral blood were collected in EDTA tubes from all participating individuals after obtaining their written consent. DNA was extracted using the PURGENE kit from Gentra Systems (Minneapolis, MN, USA), and stored at -20°C in aliquots until required.

### Determination of CAD risk factors

Serum cholesterol and triglyceride levels were measured as routine in the main Hospital Pathology Laboratory. Triglyceride levels >1.8 mmol/L and total cholesterol levels > 5.2 mmol/L were considered elevated. Diabetic patients either had a known history of diabetes mellitus or were diagnosed as such according to the American Diabetes Association criteria [13]. Diagnosis of myocardial infarction was based on the consensus specified by the European Society of Cardiology and the American College of Cardiology [14]. Body mass index (BMI) was determined for all participants, and individuals with BMI ≥ 30 were considered obese in accordance with the Center for Disease Control and Prevention (Atlanta, GA, USA). Information about all other risk factors was procured either through patient interviews or by referring to their medical records.

### Detection of the S128R (A561C) polymorphism

This was carried out by polymerase chain reaction (PCR) amplification followed by *Pst*I restriction enzyme digestion. For DNA amplification, we used the forward primer 5'- AGT AAT AGT CCT CCT CAT CAT G -3' and reverse primer 5'- ACC ATC TCA AGT GAA GAA AGA G-3', designed to amplify a 186 bp fragment of the E-selectin gene [6,11]. Each 25 μl PCR reaction contained 2.5 μl of 10X reaction buffer with MgCl_2 _(Amersham Pharmacia Biotech, Piscataway, NJ, USA), 10 ρmol of each primer, 100 ρmol/μl each of deoxynucleoside triphophates (deoxyadenosine triphosphate, deoxyguanosine triphosphate, deoxycytidine triphosphate and deoxythymidine triphosphate) (Perkin-Elmer Corporation, Foster City, CA, USA) in Tris HCl buffer, 1 unit Taq DNA polymerase (Amersham Pharmacia Biotech, Piscataway, NJ, USA) and 50 ng genomic DNA template. The mixture was denatured at 95°C for 5 min and the PCR reaction was carried out for 35 cycles, in a GeneAmp 9600 PCR system (Perkin-Elmer Corporation, Foster City, CA, USA), under the following conditions: denaturation at 95°C for 1 min, annealing at 54°C for 45 sec, extension at 72°C for 1 min and final extension cycle of 72°C for 7 min. The PCR products were electrophoresed on a 1% agarose gel and detected with 0.5 μg/ml ethidium bromide to confirm the correct amplicon size. The products were digested using the *Pst*I restriction enzyme (Stratagene, LaJolla, Calif, USA) and the resultant fragments resolved on a 4% MetaPhor agarose gel (FMC Bio-products, Rockland, Maine, USA) in TE buffer containing 0.5 μg/ml ethidium bromide. The sizes of the digested amplicons were determined using the 50-bp ladder (Amersham Pharmacia Biotech, Piscataway, NJ, USA). As a quality control, we confirmed by direct sequencing the genotype status of 384 random samples representing the three different genotypes.

### Statistical analysis

Genotype frequencies in various groups were compared by Chi-Square test. Multivariable logistic regression was used to study the effect of the E-selectin 561 C allele (128R allele) on CAD status, incorporating other variables (coronary risk factors) into the model. Additionally, we tried multiple logistic regression models involving the inclusion of interaction of the genotype × different CAD risk factors. All analyses were performed using SPSS v.10 (SPSS Inc., Chicago, USA) statistical analysis software. A two-tailed *p *value < .05 was considered statistically significant. We did power analysis employing the nQuery Adviser version.4 using two scenarios described in the results section and we concluded from this analysis that the study was adequately powered.

## Results

In the angiographically confirmed CAD patients (n = 556), 451 (81.1%) were homozygous S/S genotype, 92 (16.6%) were heterozygous S/R and 13 (2.3%) were homozygous for the R/R (Table 1). Among the CON group (n = 237), 208 (87.8%) were S/S, 28 (11.8%) were S/R and 1 (0.4%) was R/R genotype. When we compared the prevalence of the different genotypes between the CAD and CON groups, the odds ratio for the S/R genotype was 1.52 (95% CI: 0.94 – 2.45; *p *= .07) and that for the R/R genotype was 6.0 (95% CI: 0.82 – 123.6; *p *= .05).

**Table 1:** Crude odds ratio for all CAD risk factors among CAD and CON groups

**Risk factor**	***Status***	**Total**	**CAD**	**CON**	**Odds ratio**	**95% C.I**	***P *value**
Genotype	S/S	659	451	208	-	-	-
	S/R	120	92	28	1.52	0.94 – 2.45	.07
	R/R	14	13	1	6.0	0.82 – 123.6	.05
Alleles	S	1438	994	444	-	-	-
	R	148	118	30	1.76	1.14 – 2.72	.007
Age	< 40	207	139	68	-	-	-
	≥ 40	586	417	169	1.20	0.86 – 1.69	.29
Hypercholesterolemia	No	252	73	179	-	-	-
	Yes	541	483	58	20.4	13.8 – 30.0	<.001
DM	No	382	194	188	-	-	-
	Yes	411	362	49	7.16	4.9 – 10.2	<.001
FH	No	544	376	168	-	-	-
	Yes	249	180	69	1.1	0.84 – 1.62	.40
Gender	F	292	160	132	-	-	-
	M	501	396	105	3.11	2.3 – 4.3	<.001
Hypertension	No	120	49	71	-	-	-
	Yes	673	507	166	4.42	2.95 – 6.62	<.001
MI	No	599	368	231	-	-	-
	Yes	194	188	6	19.7	8.5 – 45.1	<.001
Obesity	No	73	45	28	-	-	-
	Yes	720	511	209	0.65	0.39 – 1.08	.07
Smoking	No	667	462	205	-	-	-
	Yes	126	94	32	1.30	0.84 – 2.01	.25
Hypertriglyceridemia	No	247	90	157	-	-	-
	Yes	546	466	80	10.2	7.1 – 14.4	<.001

DM, diabetes Mellitus; FH, family history of CAD; MI, myocardial infarction on admission; hypertriglyceridemia (>1.8 mmol/L); hypercholesterolemia (> 5.2 mmol/L) and obesity (Body Mass Index ≥ 30).

We found that the mutant 128R allele accounted for 11% in the CAD group, which was significantly higher than that in the CON group (6%). The odds ratio for the risk of CAD associated with the 128R allele was 1.76 (95% CI 1.14 – 2.72; *p *= .007), thus indicating a significant association of this allele with CAD in our population.

The variables showing an association (*p *=< .05) from Table 1, were then put into a stepwise logistic regression, in order to study the possible combined effect of the mutant 128R allele with other risk factors on angiographic CAD. The variables retained in the model were hypertension (*p *= .03), diabetes mellitus (DM) (*p *=< .001), hypercholesterolemia (*p *=< .001), hypertriglyceridemia (*p *= .008), MI (*p *=< .001) and gender (*p *=< .001) (Table 2).

**Table 2:** Results of multiple logistic regression analysis: final significant variables in equation

**Risk Factor (variables)**	**Odds ratio**	**[95% C.I]**	***p *value**
Hypertension	1.53	1.03 – 2.26	.03
DM	3.95	2.85 – 5.48	<.001
Hypercholesterolemia	7.96	5.30 – 11.9	<.001
Hypertriglyceridemia	1.72	1.15 – 2.58	.008
MI	14.1	7.45 – 26.7	<.001
Gender	2.36	1.73 – 3.23	<.001

As for the power calculation, we did power analysis employing nQuery Adviser version 4 using two scenarios as follow: First, In our study, when we compared the risk of CAD among (S/R and R/R) to S/S, we got an odds ratio of 1.67 (p = .023). When α =.05, using 2 sided test, proportion of S/R and R/R among CAD is .189 and among controls is .122. Number of controls = 237, number of cases = 556 (we used the average, which is 397). Using this information we have calculated the power in nQuery as 74%. Second, In our study, when we analyzed the risk of CAD among (R alleles) compared to S alleles, we got an odds ratio of 1.7 (p = .007). When α = .05, using 2 sided test, proportion of C alleles among CAD is .106 and among controls is .063. Number of controls = 474, number of cases = 1112 (we used the average, which is 793). Using this information, we have calculated the power in nQuery as 86%. From this power calculation, we concluded that our study is adequately powered.

## Discussion

In the past 3 decades, the incidence of CAD is on the rise in Saudi Arabia. According to the biggest study conducted so far, the overall prevalence of this disease in Saudi Arabia is 5.5% [15]. The rise in the incidence of CAD has been attributed to the major changes in the life-style of the Saudi population. High-fat diets, obesity, diabetes, and smoking, all of which are considered CAD risk factors, have become more prevalent, and people are leading a more sedentary lifestyle. Two independent studies reported predicted an incremental scale for the development of CAD in the Saudi population because of sharp increases in CAD risk factors such as obesity, hypercholesterolemia, diabetes, hypertriglyceridemia, and high blood pressure [16,17]. Early detection of individuals genetically susceptible to CAD can lead to early intervention, and knowledge of genetic susceptibility to CAD has value in providing risk information and guiding decision-making. To our knowledge, this study is the first to evaluate the prevalence of E-selectin polymorphism (S128R) and its potential relevance for angiographic CAD and its associated risk factors in the Arab population. The first step was to determine the prevalence of different E-selectin genotypes among our general population of Saudi Arabs. We found that the S/S is the most abundant and R/R the least common genotype among Arabs. The prevalence of the 128R allele was 6% in our controls, which is almost similar to the rate observed in the Germans (7.1%) and Japanese (6.7%) and slightly higher that in Africans (3.7%) [9] and Chinese (0%) [10]. When we compared the genotype frequencies in CAD versus CON group, the odds ratio was significant, indicating an association between these genotypes and CAD.

However, possibly because of the relatively small number of individuals with the S/R and R/R genotype, this analysis did not attain significance see Table 1. On the other hand, a significant odds ratio and *p *value was obtained when we compared the frequency of the 128R mutant allele in CAD and CON groups, pointing to an association of this allele with CAD among our Saudi Arab population. These results are comparable to the findings in the Japanese [4], German [11] white American [6] and Chinese [10] populations, but in contrast to a previous study in CAD patients with type 2 DM [12]. Although the frequencies of several classical risk factors for CAD, including elevated cholesterol and triglycerides levels, DM, age, hypertension, gender and MI were higher in the CAD patients compared to controls, when we entered these risk factors with the mutant 128R allele into a multiple variable logistic regression, the association was no longer significant.

## Conclusion

In summary, the mutant 128R allele of the E-selectin gene is associated with angiographic severe CAD in Saudi Arabs. This association is lost after adjustment for traditional CAD risk factors.

## Abbreviations

**BMI **Body Mass Index

**CAD **Coronary artery disease

**CI **Confidence interval

**CON **Control group

**DM **Diabetes mellitus

**FH **Family history of CAD

**MI **Myocardial infarction

**PCR **Polymerase chain reaction

**R **Arginine

**S **Serine

**TG **Triglycerdies

## Competing interests

The author(s) declare that they have no competing interests.

## Authors' contributions

KKA was in charge of design and analysis of data, OMA performed the technical aspect of the study, PCR and genotyping, GHM performed the statistical analysis and ND was responsible for recruiting patients and overall supervision of the study.

## Pre-publication history

The pre-publication history for this paper can be accessed here:


